# The Genetic Signatures of Noncoding RNAs

**DOI:** 10.1371/journal.pgen.1000459

**Published:** 2009-04-24

**Authors:** John S. Mattick

**Affiliations:** Australian Research Council Special Research Centre for Functional and Applied Genomics, Institute for Molecular Bioscience, University of Queensland, St Lucia, Australia; Massachusetts General Hospital, Howard Hughes Medical Institute, United States of America

## Abstract

The majority of the genome in animals and plants is transcribed in a developmentally regulated manner to produce large numbers of non–protein-coding RNAs (ncRNAs), whose incidence increases with developmental complexity. There is growing evidence that these transcripts are functional, particularly in the regulation of epigenetic processes, leading to the suggestion that they compose a hitherto hidden layer of genomic programming in humans and other complex organisms. However, to date, very few have been identified in genetic screens. Here I show that this is explicable by an historic emphasis, both phenotypically and technically, on mutations in protein-coding sequences, and by presumptions about the nature of regulatory mutations. Most variations in regulatory sequences produce relatively subtle phenotypic changes, in contrast to mutations in protein-coding sequences that frequently cause catastrophic component failure. Until recently, most mapping projects have focused on protein-coding sequences, and the limited number of identified regulatory mutations have been interpreted as affecting conventional *cis*-acting promoter and enhancer elements, although these regions are often themselves transcribed. Moreover, ncRNA-directed regulatory circuits underpin most, if not all, complex genetic phenomena in eukaryotes, including RNA interference-related processes such as transcriptional and post-transcriptional gene silencing, position effect variegation, hybrid dysgenesis, chromosome dosage compensation, parental imprinting and allelic exclusion, paramutation, and possibly transvection and transinduction. The next frontier is the identification and functional characterization of the myriad sequence variations that influence quantitative traits, disease susceptibility, and other complex characteristics, which are being shown by genome-wide association studies to lie mostly in noncoding, presumably regulatory, regions. There is every possibility that many of these variations will alter the interactions between regulatory RNAs and their targets, a prospect that should be borne in mind in future functional analyses.

## Introduction

Genome sequencing projects have shown that the numbers of protein-coding genes and the extent of protein-coding sequences do not change appreciably across the vertebrates nor indeed across the metazoa as a whole, despite large differences in developmental complexity [1]. On the other hand, the extent of non–protein-coding intronic and intergenic sequences in genomes does increase with developmental complexity, suggesting that these sequences may contain increasingly elaborate regulatory information [1].

**Figure 1:**
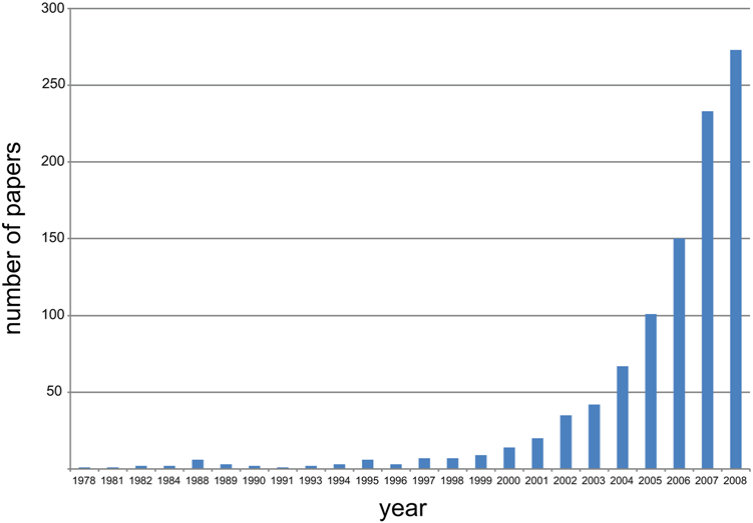
The recent rise in papers on ncRNAs. The number of indexed Medline entries with the words “non-coding RNA”, “noncoding RNA”, “non-protein-coding RNA” or “ncRNA” in the title, abstract or keywords is plotted per year. Data courtesy of Ryan J. Taft.

In recent years it has also become evident that the vast majority of the mammalian genome, and that of other complex organisms, is transcribed, apparently in a developmentally regulated manner, to produce large numbers of ncRNAs that are antisense, intergenic, interleaved, or overlapping with protein-coding genes [2]–[5]. In addition, there are increasing reports (Figure 1) of the functionality of individual ncRNAs in mammals (Table 1), other animals (see, e.g., [6]–[8]), plants (e.g., [9],[10]), and fungi (e.g., [11]), particularly in relation to developmental processes [12]. These include the involvement of ncRNAs in the regulation of the expression of homeotic genes [7],[13], oncogenes [14], and metabolic genes [15], as well as in the regulation of skeletal development [16], eye development [17], epithelial-to-mesenchymal transition [18], and subcellular structures [19]–[22], among many others (for reviews and additional examples, see [4], [12], [23]–[25]).

**Table 1:** Examples of functional mammalian noncoding RNAs.

Name	Characteristics	Function	Experimental Methodology^a^	References
Air	108 kb; transcribed from an antisense promoter located in intron 2 of Igf2r	Regulates genomic imprinting of a cluster of autosomal genes on mouse chromosome 17	Mutagenesis; FISH; ChIP; RNA-IP	[156],[189],[196]
BACE1AS	∼2 kb; transcribed antisense to beta-secretase-1 (BACE1) gene, elevated in Alzheimer disease	Regulates BACE1 expression in vitro and in vivo, influences amyloid-beta 1–42 levels	shRNA and siRNA knockdown	[197]
BC1	152 nt; expressed by a specific subset of neurons in the central and peripheral nervous system; dendritic location	Affects exploratory behaviour and anxiety, represses translation by targeting initiation factor 4A helicase	In vivo knockout, biochemical analyses	[100],[198]
Borg	∼2.8 kb; induced by bone morphogenic proteins (BMPs) and osteogenic proteins	Regulates BMP-induced differentiation of C2C12 cells into osteoblastic cells	Antisense knockdown	[199]
CCND1 associated ncRNAs	Unspecified sizes; transcribed from the promoter region of the cyclin D (CCND1) gene; induced by DNA damage signals	Allosterically modifies the RNA-binding protein TLS (“translocated in liposarcoma”), leading to inhibition of CREB-binding protein and histone acetyltransferase activities to repress cyclin D1	ChIP; RNA-IP; siRNA knockdown	[200]
CUDR	∼2.2 kb; up-regulated in a doxorubicin-resistant human squamous carcinoma	Regulates drug sensitivity, cellular transformation and apoptosis	Overexpression	[201]
EGO	∼1.0 and 1.7 kb; highly expressed in human bone marrow and in eosinophil development	Regulates expression of myelin basic protein and eosinophil-derived neurotoxin mRNAs	siRNA knockdown	[202]
DHFR upstream	Unspecified size; transcribed upstream of the dihydrofolate reductase (DHFR) gene	Regulates DHFR expression by formation of triple helix in the DHFR promoter	siRNA knockdown,; ChIP; RNA-IP, other	[15]
Evf-2	∼3.8 kb; antisense to *Dlx6*; developmentally regulated, expressed in the ventral forebrain	Cooperates with Dlx-2 in vivo to increase the transcriptional activity of the Dlx-5/6 enhancer in a target and homeodomain-specific manner	Overexpression; siRNA knockdown; mutagenesis; ChIP	[154]
Gadd7	754 nt; induced by lipotoxic-stress	Regulates lipid-induced oxidative and ER stress	Mutagenesis; shRNA knockdown	[203]
GAS5	∼7 kb; growth arrest-specific transcript, multiple splice isoforms, encodes several snoRNAs in its introns, down-regulated in breast cancer	Controls apoptosis and the cell cycle in lymphocytes	siRNA knockdown; overexpression	[204]
H19	2.3 kb; imprinted (maternal allele active) at the *Igf2* locus; strongly expressed during embryogenesis; up-regulated in various tumours	Complex functions, influences growth by way of a cis control on Igf2 expression implicated as both a tumour suppressor and an oncogene	siRNA knockdown; overexpression; in vivo knockout	[205]–[207]
HOTAIR	2.2 kb; transcribed from the *HOXC* locus from a position intergenic and antisense to the flanking *HOXC11* and *HOXC12* genes	Epigenetically silences gene expression at the *HOXD* locus	siRNA knockdown	[13]
HOTAIRM1	483 nt; specific to the myeloid lineage	Involved in RA-induced expression of HOXA1 and HOXA4 during myeloid differentiation, and induction of myeloid differentiation genes *CD11b* and *CD18*	shRNA knockdown	[49]
Hsr1	∼600 nt; ubiquitously expressed	Required for heat shock response	siRNA and antisense knockdown	[208]
IGS RNAs	150–300 nt; originate from the intergenic spacers (IGS) that separates rRNA genes; bind to the chromatin remodelling complex NoRC	Required for the nucleolar localization of NoRC and epigenetic control of the rDNA locus	Mutagenesis; antisense knockdown	[209]
Kcnq1ot1	91 kb; paternally expressed from *Kcnq1* imprinting control region	Mediates organization of a lineage-specific nuclear domain involved in epigenetic silencing of the *Kcnq1* imprinting control region	Mutagenesis; ChIP; RNA/DNA FISH	[22],[155],[157],[210]
Khps1a	1,290 nt; originates from the CpG island and overlaps a tissue-dependent differentially methylated region of Sphk1	Regulates DNA methylation in the tissue-dependent differentially methylated region of Sphk1	Overexpression	[211]
lincENC1	Size unspecified; ∼181 kb from Enc1	Regulates cell proliferation in embryonal stem cells	shRNA knockdown	[32]
MEG3	∼1.6 kb; maternally expressed from the Dlk1-Gtl2 imprinted locus	Regulates p53 expression, inhibits cell proliferation in the absence of p53	Overexpression, mutagenesis	[192]
MEN ε/β (Neat1)	∼3.5 and ∼23 kb; up-regulated upon muscle differentiation; transcribed from the multiple endocrine neoplasia 1 (MEN1) locus	Required for the structural integrity of nuclear paraspeckles	Antisense, siRNA knockdown; overexpression; FISH	[19]–[21]
ncR-uPAR	∼350 bp; upstream of human protease-activated receptor-1 (PAR-1) gene	Up-regulates Par-1 promoter	Overexpression	[212]
Nkx2.2AS	4.3 kb; antisense to Nkx2, preferentially expressed in the nervous system	Enhances oligodendrocytic differentiation	Overexpression	[213]
NRON	∼2.7 kb; enriched in placenta, muscle, and lymphoid tissues	Modulates NFAT nuclear trafficking	siRNA knockdown	[214]
p15AS	38.4 kb; antisense to the tumour suppressor gene p15	Epigenetically silences p15 expression	Overexpression	[14]
PCGEM1	1,643 nt; prostate tissue-specific and prostate cancer-associated	Inhibits apoptosis induced by doxorubicin	Overexpression	[215]
PRINS	∼3.6 kb; elevated expression in psoriatic epidermis; regulated by the proliferation and differentiation state of keratinocytes.	Required for cell viability after serum starvation	siRNA knockdown	[42]
PINC	∼1 and 1.6 kb; developmentally regulated, expressed in mammary gland	Performs dual roles in cell survival and regulation of cell-cycle progression	siRNA knockdown; FISH	[216]
RepA	∼1.6 kb; internal to *Xist*	Recruits the Polycomb complex, PRC2, to the inactive X chromosome, with Ezh2 serving as the RNA binding subunit	ChIP; FISH; overexpression; shRNA knockdown	[128]
SAF	1.5 kb; transcribed from the opposite strand of intron 1 of the human Fas gene	Regulates Fas-mediated but not TNF-alpha-mediated apoptosis	Overexpression	[217]
SatIII	Various sizes up to >1.4 kb; transcribed from satellite DNA associated with, and localized in, nuclear stress bodies	Mediates recruitment of RNA processing factors to, and formation of, nuclear stress bodies	Antisense and siRNA knockdown	[218]
SCA8	Predicted gene product underpinning the triplet repeat expansion-induced neurodegenerative disease Spinocerebellar Ataxia 8	Induces late-onset, progressive neurodegeneration in the Drosophila retina; associates with the RNA binding protein staufen	Ectopic expression in *Drosophila*; genetic modifier screen	[39]
TERRA / TelRNAs	Various sizes; transcribed from and associated with telomeres; contain UUAGGG repeats	May form G-quartet structures with telomere DNA; inhibit telomerase activity	RNA FISH; oligo-nucleotide inhibition	[219],[220]
Tsix	∼40 kb; antisense to Xist	Epigenetically silences *Xist* expression by inhibiting RepA recruitment of polycomb complexes to maintain the active X chromosome in females	Mutagenesis	[128], [221]–[223]
TUG1	6.7 kb; expressed in the developing retina and brain	Required for the proper formation of photoreceptors in the developing rodent retina	shRNA knockdown	[17]
UCA1	1,442 nt; expressed in embryonic development and bladder cancer	Enhances tumorigenic behaviour of bladder cancer cells in vitro and in vivo	Overexpression	[224]
Xist	∼17 kb; mosaic expression in females	Epigenetically controls dosage compensation by silencing one of the two X chromosomes	Mutagenesis	for recent review see [99]
Y RNAs	83–112 nt; up-regulated in cancer; bound by Ro autoantigen	Regulate cell DNA replication and cell proliferation	siRNA knockdown	[47],[225]
Zeb2NAT	>680 nt; antisense to Zeb2, a transcriptional repressor of E-cadherin	Regulates splicing of the Zeb2 5′ UTR	Overexpression	[226]
Zfh-5AS	∼10 kb; expressed in particular regions of the developing brain	Regulates expression of the transcription factor Zfh-5 mRNA	Mutagenesis	[227]

This list is not exhaustive, and there are other examples of functional ncRNAs in mammals (see e.g. [228]) as well as of regulatory and structural ncRNAs in other animals, plants, fungi (see e.g. [6]–[11]) and bacteria [229].

a Abbreviations used are: siRNA, short interfering RNA; shRNA, short hairpin RNA; FISH, fluorescence in situ hybridization; ChIP, chromatin immunoprecipitation; RNA-IP, immunoprecipitation of RNA associated with particular proteins.

There is also compelling genome-wide evidence that the large numbers of identified but as yet unstudied intronic, intergenic, and antisense ncRNAs have intrinsic indices of functionality (Table 2), as indicated by the following: (i) the conservation of their promoters, splice junctions, exons, predicted structures, genomic position, and expression patterns [2], [26]–[36]; (ii) their dynamic expression and alternative splicing during differentiation [13],[31],[32]; (iii) their altered expression or splicing patterns in cancer and other diseases [37]–[49]; (iv) their association with particular chromatin signatures that are indicative of actively transcribed genes [31],[32]; (v) their regulation by key morphogens and transcription factors [31],[32],[49],[50]; and (vi) their tissue- and cell-specific expression patterns and subcellular localization [16], [17], [19]–[22], [49], [51]–[56].

**Table 2:** Indices of the functionality of ncRNAs.

Feature	References
Conservation of promoters	[2],[27],[32]
Conservation of splice junctions	[27]
Conservation of sequence	[26],[27],[32]
Conservation of genomic position	[31],[33],[34]
Conservation of secondary structure	[28]–[30]
Positive selection	[230]
Conservation of expression	[35],[36]
Dynamic expression and alternative splicing	[13],[31],[32]
Altered expression or splicing in cancer and other diseases	[37]–[49]
Association with particular chromatin signatures	[31],[32]
Regulation by morphogens and transcription factors	[31],[32],[49],[50]
Tissue- and cell-specific expression patterns	[16], [17], [19]–[22], [49], [51]–[56]
Specific subcellular localization	[19]–[22],[52],[56]

Indeed, a recent study of over 1,300 mouse ncRNAs showed that almost half of them exhibit precise expression patterns in different parts of the brain, including different subregions of the hippocampus, olfactory bulb, neocortex, and cerebellum [52]. While many ncRNAs, like other regulatory sequences (see below), appear to be evolving quickly, including those with well-validated functions [26], most retain conserved patches within them [26],[32], and some may be under positive selection, especially in the brain [57]. At least some of these RNAs, including intronic RNAs, are precursors for small regulatory RNAs like microRNAs (miRNAs), small interfering RNAs (siRNAs), piwi-interacting RNAs (piRNAs), and small nucleolar RNAs (snoRNAs) [58]–[68]. Many miRNAs are highly conserved from nematodes to humans, whereas others are primate-specific, and their full repertoire remains to be determined [4],[69],[70].

It is widely accepted that animals have a relatively common set of protein-coding genes and that, notwithstanding lineage-specific innovations and splice variants, the primary basis of phenotypic, especially morphological, radiation and higher complexity has been the variation and expansion of the regulatory architecture that controls the deployment of these protein components and their isoforms during differentiation and development [71]. This regulatory architecture is generally more plastic than protein-coding sequences that are highly constrained by relatively strict structure-function relationships, which is reflected by the fact that regulatory sequences evolve at widely different rates [72]–[75]. These sequences range from promoter regions that have no recognizable sequence similarity yet direct orthologous patterns of gene expression between fishes and mammals [76] to highly conserved non-genic elements [77],[78], and “ultraconserved” sequences that have remained essentially unchanged over hundreds of millions of years of vertebrate evolution and appear to act as tissue-specific enhancers that regulate gene expression during development [79]–[82].

Regulatory sequences are also generally assumed to operate through their interactions with sequence-specific transcription factors and other regulatory proteins, but this assumption has been made in ignorance, until recently, of the extent of developmentally regulated transcription of ncRNAs from the genome, including many regions spanning enhancers and promoters (see, e.g., [45], [83]–[86]). The possibility is therefore that the genomes of mammals and other complex organisms encode a large repertoire of regulatory RNAs [4]. Indeed, the case has been made that a much higher degree of regulatory sophistication, aided by the co-option of the considerable powers of RNA to transmit sequence-specific information, was a prerequisite for the evolution of developmentally complex organisms [87],[88], and that many of these RNAs may be involved in the regulation of developmental processes, including the epigenetic trajectories that underpin them, for which there is increasing evidence [12],[89].

However, if these ncRNAs are functional and important in developmental and physiological processes, why have so few been identified in genetic screens to date? Here I outline the emerging evidence for ncRNA involvement in key molecular genetic phenomena and in specific functions and phenotypes. I also outline the expectational, perceptual, and practical factors that may collectively account for the low genetic visibility of individual ncRNAs. Awareness of these factors and the possibility that the structure of the genomic programming of complex organisms is different from our current understanding may lead to the increased recognition of ncRNAs in genetic analyses, assisted by the emerging fusion of genetics, genomics, and systems biology.

## Phenotypic Impact

The ability to detect a relevant mutation or variation is dependent on the sensitivity of the phenotypic screen. Mutations in protein-coding sequences usually give severely compromised (i.e., obvious) phenotypes, whereas those in regulatory sequences often do not. Proteins are the key structural and functional analogue components of cells, and the loss of their function is often disastrous, leading in many cases to obvious defects, and in some cases to embryonic lethality. Mutations in generic transcription factors and other “regulatory” proteins are included, and their loss causes pleiotropic effects on gene expression at many loci and plays an important role in the molecular etiology of cancer [71],[90],[91]. This is in contrast to regulatory sequences, which, when damaged, may only affect a small part of the network, with more restricted and subtle consequences, often referred to as quantitative trait variations. Indeed the use of the word “mutation”, as opposed to “variation”, reflects an inherent bias in the identification of genetic factors that influence phenotype in animals, with those exhibiting strong effects understandably having taken precedence over those that do not, both perceptually and practically. Consistent with this, until recently, there were relatively few regulatory mutations identified among the catalogue of known human mutations that are associated with overt genetic disease.

**Figure 2:**
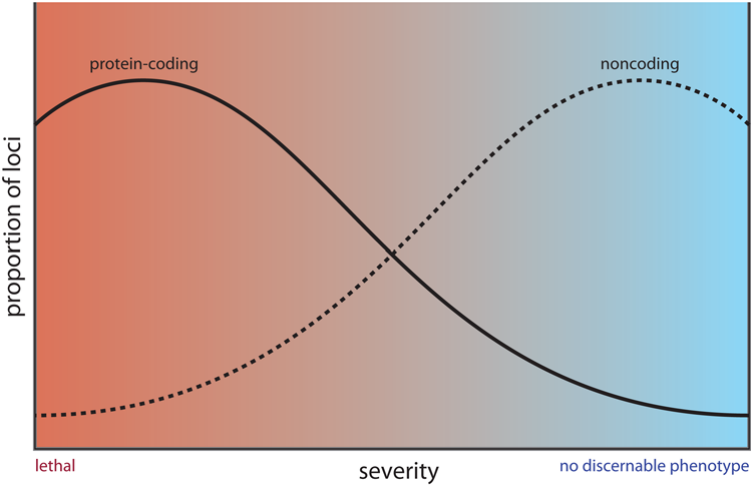
The contrasting effects of mutations in protein-coding and regulatory sequences. A conceptual diagram of the spectrum of phenotypic effects of mutations in sequences encoding proteins and other analogue components of cells (continuous line) versus variations in non-coding sequences that specify regulatory interactions (dashed line).

There is, of course, a wide spectrum of effects of both coding and noncoding mutations (Figure 2), and there are exceptions to the rule in both directions. Loss-of-function mutations in some protein-coding genes have mild effects [92], as exemplified by as knockouts of the mammalian genes encoding calbindin D9k [93] and C/EBPdelta [94],[95], and the significant number of yeast genes that show no observable phenotype. Reciprocally, knockouts of some highly conserved miRNAs, many of which have multiple targets, give strong phenotypes [96]–[98], even though very few such genes have been identified in genetic screens in *Caenorhabditis elegans* and *Drosophila*, and none have been identified in mice, despite the intensity of such screens (see below). Moreover, to date, no naturally arising mutations have been discovered in the *Xist* gene in either humans or mice, despite the central role that this ncRNA plays in embryogenesis and in X-chromosome dosage control in females [99], possibly because such mutations are lethal.

On the other hand, the potential subtlety of mutations in ncRNAs, even when they are targeted by reverse genetics, is illustrated by the case of *BC1*, which is expressed in synaptodendritic domains of neurons in rodents. Knockout of this transcript produces no obvious physical or neurological abnormality, and the mutant mice were initially indistinguishable from wild type, but were subsequently found to have reduced exploratory behaviour and consequently a higher mortality in field experiments [100]. Thus this ncRNA causes a subtle behavioural phenotype that is invisible in the cage, and would escape detection in superficial forward genetic screens, but is almost certainly strongly disadvantageous in the wild. Similarly, deletions or insertions in some ultraconserved enhancers yield no discernable abnormality [101],[102], despite the fact that these sequences are clearly under intense selection [103], suggesting insensitivity of phenotypic screens in captivity or redundancy in the regulatory architecture (perhaps associated with developmental robustness) that we have yet to understand.

## Monogenic Diseases and High Penetrance Mutations

The generally stronger effects of protein-coding mutations leads to a sampling bias, in that more severe phenotypes are not only more easily discerned but have also been more likely to attract further study, both in medical contexts and in model organisms. In medicine, mutations involving catastrophic component damage have been traditionally referred to as “monogenic diseases” and were the primary targets of study in the pioneering days of human genomics, just a decade or two ago, when the protein-coding genes underpinning cystic fibrosis, Huntington disease, and Duchenne muscular dystrophy, among others, were identified by positional mapping and cloning approaches. Thus, in humans, mutation mapping is still both a young science and extraordinarily difficult due to the sheer complexity of the genome, which naturally led to an initial focus on severe loss-of-function diseases that exhibit simple inheritance patterns with high penetrance (“single gene–large effect”) making them amenable to identification. This is now changing with the increasing availability of human genome sequences, and the application of genome-wide association (GWA) studies to the mapping of the genetic components of complex traits, wherein lies the majority of the health burden and the majority of the interesting phenotypic differences between individuals and species.

## Complex Traits and GWA Studies

Indeed, functional classification of sequence variations that have been identified by GWA studies to be associated with complex traits, albeit still strongly focused on those of medical importance, shows that the vast majority of variations are located in noncoding regions [104],[105]. In most cases, the causative mutations have yet to be defined, and their mechanistic basis is unknown—especially whether they affect *cis*-acting binding sites for regulatory proteins or the function or expression of regulatory RNAs. One good candidate for the latter is the ncRNA *ANRIL*, which lies antisense to *CDKN2A* and traverses a noncoding region centromeric to *CDKN2A*, a region implicated in a range of complex diseases including cancer, type 2 diabetes, periodontitis, and coronary heart disease [106]–[112]. Perplexingly, however, those variants mapped by GWA studies to date account for only a small proportion of genetic variation in disease or quantitative traits [113]. These traits are clearly multifactorial and may be affected by rare variants with strong effects that have yet to be recognized.

The identification of the sequence changes that directly underpin quantitative trait variations has thus far been possible, or at least achieved, only in well-structured pedigrees in plants and animals. In the few cases where such quantitative trait loci (QTLs) have been mapped to completion, most have been found to be located in noncoding sequences, specifically: (i) regulatory sequences in promoters and distal enhancers (e.g., the “*teosinte branched1*” mutation affecting branching and inflorescence in maize [114]); (ii) 3′ untranslated regions (UTRs) (e.g., those underlying Tourette's syndrome [115], and muscular hypertrophy in sheep [116]; see also below); (iii) introns (e.g., a QTL affecting muscle growth in domestic pigs [117]); or (iv) intergenic sequences of unknown transcriptional status (e.g., the “*callipyge*” mutation causing posterior muscular hypertrophy in sheep [118]). The latter occurs in an imprinted locus and affects the expression of a number of protein-coding and ncRNA genes [119] associated with an unusual genetic phenomena termed “polar overdominance” [120], which may also occur in humans [121]. While these mutations are reasonably assumed to be regulatory in nature, their mechanistic basis has not been determined, although in the latter case, there is some evidence for the involvement of *trans*-acting miRNAs [119],[122]. In addition, linkage studies in a large family have recently identified the ncRNA *AK023948* as a candidate susceptibility gene for papillary thyroid carcinoma [123].

## Type of Mutation and Sensitivity of the Model System

The nature of the organism under study and the type of mutations also affect the outcome of genetic screens—relevant single-base mutations are not only harder to identify than insertions/deletions, especially in mammalian genomes and even in inbred mice, but also have milder effects on regulatory sequences.

Most mutations induced by ENU mutagenesis involve single-base changes, which are also, along with small indels and copy number variations, the most common type of natural variation in humans and other mammals, where few regulatory mutations have yet been identified. Whereas a nonsynonymous mutation in a protein-coding sequence can have severe effects on the structure and function of the protein, many regulatory sequences have loose consensus sequences, and variations in them, as noted already, may have subtle effects and go unnoticed, especially in superficial phenotypic screens.

On the other hand, insertions and deletions dominate mutational screens in *Drosophila*, and a large number map to noncoding intergenic and intronic regions. This is exemplified by the intensively studied *bithorax* complex, where there are not only mutations known in the coding sequences for the homeotic protein Ultrabithorax (Ubx), but also in upstream (*bithoraxoid* or *bxd*) and intronic (*Contrabithorax* or *Cbx* and *anterobithorax* or *abx*) sequences [124], which contain conserved blocks within them [125]–[127]. Such noncoding mutations are interpreted as affecting orthodox *cis*-acting enhancer sequences (i.e., those that bind *cis*-acting regulatory proteins), despite the fact that (for example) *bxd* mutations fall within a region that is transcribed during early embryogenesis into a complex set of short polyadenylated RNAs with no coding potential. These RNAs arise by alternative splicing of at least 11 exons derived from a 26-kb primary transcript [126]. Moreover, these regulatory regions involve interaction with Polycomb-group and Trithorax-group proteins, which are increasingly implicated as being directed to their sites of action by ncRNAs [7],[13],[128] (see below).

Similarly the *iab* regulatory elements of the bithorax complex that control the expression of *abdominal-A* and *Abdominal-B*, and consequently the identities of the 2nd–9th abdominal segments, are transcribed into ncRNAs in a spatially ordered pattern [129]. It has also recently been shown that 231 ncRNAs are expressed from the four human *HOX* loci in a spatially and temporally ordered progression along developmental axes, one of which (termed *HOTAIR*) from the *HOXC* locus controls expression of the *HOXD* locus in *trans*, as shown by siRNA-mediated knockdown experiments [13]. None of these ncRNAs has yet been specifically associated with a genetic variant in mammals, although in *Drosophila* there are many homeotic mutations that lie in regions encompassed by ncRNAs [12],[124], and *Drosophila* geneticists were clearly intrigued by them [130]. Moreover, as pointed out by Rinn et al. [13], the existence of such transacting regulatory ncRNAs may explain the observation that the deletion of the entire *HOXC* locus exhibits a milder phenotype than the deletion of individual *HOXC* genes [131].

Despite the now known importance of miRNAs in the control of gene expression [67],[132], only four miRNA loci have been identified in intense genetic screens in *C. elegans* and *Drosophila*, and none in mammals. This may be partly due to the fact that *C. elegans* is hermaphroditic and naturally driven to homozygosity in individual isolates, without the need for laborious back-crossing, and flies are routinely bred to homozygosity in mutational screens, making screening of recessive mutations far more efficient than in mice. There was also an element of serendipity, in that the first identified miRNA locus, *lin-4*, expressed a small RNA whose complementary sequence was present in multiple copies in the 3′ UTR of its target gene, *lin-14*, and thus was relatively easy to pinpoint [133],[134], which also applied in the subsequent case of *let-7* [135].

Following the discovery of miRNAs, *Drosophila* mutants of uncertain provenance that mapped in gain-of-function screens to noncoding regions were re-analysed, and one that regulates growth [136], termed *bantam*, was identified to encode a miRNA [137]. The miRNAs *let-7* [135] and *lsy-6* [138] were also identified genetically in *C. elegans*, not in other organisms, despite the former being not only highly conserved in sequence and expression pattern throughout metazoan evolution [139], but also fundamental to normal and abnormal developmental processes [140]–[142], as are many other miRNAs [143]. On the other hand, *lsy-6* is expressed in only a few cells [144], and has only rarely turned up in deep sequencing libraries. Subsequently most known miRNAs, of which there are hundreds in mammals, and later piRNAs, have been identified by biochemical not genetic means. In view of the *lsy-6* example and the clearly incomplete sampling of the small RNA transcriptome, even using deep sequencing [70], there are likely to be many more.

## Expectations and Interpretations

There has also been a strong expectation that mutations that have phenotypic effects will map to protein-coding genes or *cis*-regulatory elements that interact with regulatory proteins. The former has influenced the practical strategies for mutation searching, in terms of a focus on exon scanning of candidate genes (see below), and the latter has influenced the interpretation of regulatory variations, although in only a few cases has the mechanistic basis been determined [71],[145]. Some mutations map to gene “deserts” (see, e.g., [146]), and while it is conceivable that they affect distal enhancers (see, e.g., [147]), it is interesting and relevant to note that there is good evidence that enhancers and other regulatory sequences are transcribed into ncRNAs in the cells in which they are active [45], [83]–[86], and hence may act in part via ncRNAs.

### Transvection and Locally Acting ncRNAs

Many loci, such as the *bithorax* complex referred to earlier, exhibit a genetic phenomenon called “transvection,” whereby a wild-type regulatory region upstream of a defective protein-coding sequence on one chromosome can rescue a relatively normal phenotype, when it is combined with a mutant regulatory region linked to a wild-type protein-coding sequence on the homologous chromosome (both of which give mutant phenotypes when homozygous) [130]. This phenomenon is well documented in *Drosophila* but appears to occur in most animals and has been interpreted as a physical cross-talk between functional *cis*-acting promoters or enhancers on one chromosome to engender transcription of adjacent protein-coding genes on the other, since the effect is usually pairing-dependent and lost when the regulatory and protein-coding sequences are separated to nonsyntenic positions in the genome [130],[148]. However, this is not always the case—at some loci, transvection between regulatory elements and protein-coding sequences can operate over large distances (even between different chromosomes) [149]–[152], suggesting the involvement of a trans-acting signal. Moreover, many promoter elements that exhibit transvection are transcribed into ncRNAs, and transvection is altered in *Polycomb* and *zeste* mutants [125]–[127],[130], indicating that epigenetic processes (which may be regulated by ncRNAs, see below) are involved. Taken together, these observations raise the possibility that transvection is mediated by *trans*-acting RNAs [153], in which case the observed cross-complementation may occur simply as a result of a compound heterozygosity between a regulatory ncRNA locus and a nearby protein-coding locus whose expression is controlled by it.

In support of this proposition, there is now rapidly emerging evidence that many ncRNAs derived from either same or opposite strands act locally to regulate the epigenetic status and expression of nearby protein-coding genes, often involving the recruitment of chromatin-activator or repressor complexes [7], [13]–[16], [89], [154]–[157], with sense-antisense pairs in some cases being the substrate for the generation of siRNAs [63]–[65],[68]. Moreover the many deletion studies of gene promoter regions to define regulatory sequences have almost always assumed, physically and mechanistically, that resultant changes to expression patterns are due to the loss of *cis*-acting protein binding sites rather than deletions in the same or opposite strand ncRNAs that frequently traverse and are expressed from the same region. The complexity of these relationships is illustrated by the examples of the ncRNA *DLeu2* (*deleted in lymphocytic leukemia 2*), which has multiple splice variants and lies antisense to genes in a region deleted in various malignancies [158], and the ncRNA *ANRIL* referred to earlier. Thus the interpretation of the mechanisms by which such mutations operate remains not only an open question but a difficult problem to disentangle, given the complex interlacing and overlapping coding and noncoding transcripts, and splice variants thereof, that are expressed from many loci in different cells and tissues [2],[3].

### Transinduction, Ectopic Expression and Gene Knockouts

RNA is also implicated in a curious genetic phenomenon called “transinduction,” whereby transient transfection of a β-globin gene induces transcription of the “locus control region” and intergenic regions at the chromosomal β-globin locus in non-erythroid cell lines. This effect is dependent on transcription of the globin gene from the transfected plasmid and its association with the endogenous β-globin locus, but not on protein expression, and therefore is RNA-mediated [83], although the responsible sequences have not been mapped. Indeed, the general assumption that mRNA is simply an intermediate between gene and protein, albeit with *cis*-acting regulatory elements, may be incorrect, and there may be a false dichotomy between coding and noncoding RNA [159]. This is indicated by the complexity of overlapping sense and antisense coding and noncoding transcripts from most genomic loci [2]–[5] and evidence that protein-coding sequences are under constraint not only at the amino acid sequence level, but also within their RNA sequence [160]. Moreover, given that many gene knockout studies concomitantly delete potential sources of regulatory ncRNAs such as introns (given that many miRNAs and all snoRNAs are sourced from introns) and antisense transcribed sequences, all aspects of the observed phenotypes cannot be unequivocally or solely ascribed to the loss of the protein without complementation studies or more precise deletions that are rarely done for reasons of technical difficulty.

## Technical Limitations

The focus on protein-coding sequences has been reinforced by a practical problem, especially in mammals. Mutation mapping studies using whole-genome scanning techniques usually have not pinpointed the causative mutation or variation, and the affected region may encompass one megabase or more. Until recently, comparative sequencing of such regions was not feasible, and in any case it can be very difficult to sort the relevant polymorphism from the considerable background variation in huge intergenic and intronic regions, especially in outbred human populations. In most circumstances, understandably, investigators have resorted to analysing the most plausible candidate genes (recently including those expressing noncoding transcripts or ESTs [158],[161]) usually involving scanning of known exons (and sometimes the immediate 5′ flanking promoter sequences and UTRs) in the region by PCR amplification, in the hope that they can identify the causative mutation in these locations, which in turn are the ones that are then reported in the literature. However, there are many informal reports of mapping studies that have not identified such exonic mutations, and which consequently lie in abeyance, including (as noted already) the large number of GWA studies that have mapped disease-associated variations to noncoding, presumably regulatory, regions [104]. A reasonable strategy for searching for the relevant variations in these regions may be to focus on sequences that exhibit evolutionary conservation and/or whose expression is altered [162]. Conversely, reverse genetic screens looking for phenotypes associated with ncRNAs might target conserved blocks within them and focus on tissues where they are known to be expressed.

## Mutations Affecting *trans*-Acting Functions of mRNAs

As noted above, regulatory mutations have been identified in the 3′ UTRs of mRNAs, such as those underlying Tourette's syndrome [115] and muscular hypertrophy in sheep [116], which appear to involve gain- or loss-of-function of miRNA binding sites. Interestingly, however, a number of other reported 3′ UTR mutations do not appear to act in *cis* to regulate the expression of the associated mRNA, as is normally assumed, but rather in *trans* as ncRNAs. For example the 3′ UTR of prohibitin (in the absence of the associated protein-coding sequences) can inhibit cell cycle progression in one complementation group of breast cancer–derived cells that is characterized by naturally occurring mutations in the 3′ UTR, indicating that these sequences are in fact functioning, in part, as *trans*-acting ncRNAs [163],[164]. Similarly, the oogenesis defect observed in *Drosophila oskar* null mutants is rescued by the *oskar* 3′ UTR alone [165].

A *trans*-acting function for mRNA sequences, both coding and noncoding, may be more general that expected. For example, the introduction of cancer-associated silent point-mutations in *p53* mRNA alters its binding to the protein Mdm2, which in turn alters *p53* expression and function [166]. The 3′ UTRs of troponin I, tropomyosin, and α-cardiac actin have been shown to reactivate muscle-specific promoters in a differentiation-defective myoblast mutant, enhance the differentiation of wild-type muscle cells, and suppress the proliferation of fibroblasts independently of their normally associated protein-coding sequences [167]. Similarly, the 3′ UTRs of tropomysin [168] and ribonucleotide reductase [169] can suppress tumour formation, and the 3′ UTR of the DM protein kinase gene, which is involved in myotonic dystrophy, inhibits the differentiation of C2C12 myoblasts [170]. Moreover, many 3′ UTRs in mouse appear to be expressed separately from their mRNAs in a developmentally regulated manner [171].

There other examples of mutations in sequences encoding 3′ UTRs that do not act via the UTR. A single nucleotide polymorphism that determines susceptibility to an autoimmune thyroid disease occurs both within the 3′ UTR of the *ZFAT* gene (zinc-finger gene in AITD susceptibility region) and also within the promoter of an antisense transcript (*SAS-ZFAT*), and increases the expression of *ZFAT* not through increasing mRNA stability, but by repressing the expression of the antisense transcript [172].

## Regulation of Complex Genetic Processes by ncRNAs

Apart from the general presumption that most ncRNAs will be involved in regulation, variations in which will often have, individually, subtle effects on phenotype, there is, in fact, general evidence of their positive genetic signatures, as ncRNAs underpin most, if not all, complex genetic processes in the higher organisms. These include RNA interference-related phenomena such as co-suppression and transcriptional gene silencing [132], [173]–[176], as well as position effect variegation [177],[178], hybrid dysgenesis [179], parental imprinting, X-chromosome dosage compensation and allelic exclusion [180], germ cell reprogramming [181], and paramutation [182],[183], all of which involve epigenetic processes. Indeed, as noted already, there is increasing evidence that a major function of ncRNAs, both small and large, is the regulation of epigenetic memory through modifications to DNA and chromatin structure, involving the recruitment of DNA methyltransferases, histone-modifying enzymes, and chromatin remodelling complexes to their appropriate sites of action (including ncRNA genes themselves) in particular cells at particular stages of differentiation (for reviews, see [12],[89],[184]; also Table 1).

## Examples of Mutations in ncRNAs

There are some known examples of mutations in ncRNAs, aside from those mentioned already, that give recognizable phenotypes or that are strongly implicated in altered phenotypic states. These include a triplet repeat expansion in the ncRNA SCA8, which causes the human neurodegenerative disease Spinocerebellar Ataxia 8 (which as a transgene can induce progressive retinal neurodegeneration in *Drosophila*) [39] and other examples of deleterious gain-of-function mutations in noncoding RNAs associated with diseases such as myotonic dystrophy [185], deletions encompassing ncRNA loci and alterations to ncRNA splicing patterns in various cancers [47],[106],[158],[161],[186],[187], and a SNP variant in an ncRNA MIAT that confers risk of myocardial infarction [188]. They also include many ncRNAs, including small nucleolar RNAs, that appear to be important in the mechanism of imprinting [189] and the molecular etiology of associated pathologies such as Prader-Willi syndrome [190],[191], some that are implicated as tumour suppressors [168],[192], or that are located at chromosomal translocation breakpoints associated with B-cell lymphoma [193] and schizophrenia [194]. It has also been shown that the translocation and induced expression of an antisense, long ncRNA can cause the epigenetic silencing of the adjacent α-globin gene, resulting in α-thalassemia [195].

## Conclusion

There is not (yet) a huge catalogue of mutations in ncRNAs that have been shown to affect phenotype, compared to those in protein-coding sequences. However, on the assumption that most ncRNAs are regulatory and that most regulatory regions have yet to be assigned genetic signatures, it is no surprise that this may be the case. On the other hand, as screening for variations affecting complex traits becomes more sophisticated, it is reasonable to anticipate that many will map to, and affect the function of, ncRNAs. Certainly this possibility should be borne in mind in the interpretation of such variations and the consequent studies to define their mechanism of action. The functional analysis of ncRNAs is in its infancy, but in situ hybridization, genomic, and structural characteristics, and the perturbation of their expression by overexpression and siRNA-mediated knockdown are emerging as major tools (Tables 1 and 2). There seems little doubt that there is a hidden world of regulatory architecture underpinning the development of complex organisms that we have yet to explore, both genetically and functionally.
